# General equilibrium with endogenous trading constraints

**DOI:** 10.1371/journal.pone.0203814

**Published:** 2018-09-17

**Authors:** Sebastián Cea-Echenique, Juan Pablo Torres-Martínez

**Affiliations:** 1 School of Industrial Engineering, Pontificia Universidad Católica de Valparaíso, Valparaíso, Chile; 2 Department of Economics, Faculty of Economics and Business, University of Chile, Santiago, Chile; Universita Cattolica del Sacro Cuore, ITALY

## Abstract

In a competitive model where agents are subject to endogenous trading constraints, we make the access to financial trade dependent on prices and consumption decisions. Our framework is compatible with the existence of both credit market segmentation and market exclusion. In this context, we show equilibrium existence in two scenarios. In the first one, individuals can fully hedge the payments of segmented financial contracts by trading unsegmented assets. In the second one, it is assumed that agents may compensate with increments in present demand the losses of well-being generated by reductions of future consumption.

## Introduction

Equilibrium in incomplete markets where agents are subject to restricted participation was studied in seminal articles by [1], [2] and [3] or [4]. [1] guarantees equilibrium existence in real asset markets under exogenous short-sale constraints. [3] or [4] and [2] explore a more general framework in economies with nominal assets, considering restrictions given by closed and convex sets containing the zero vector.

Subsequent contributions to this literature have encompassed different types of constraints. Linear equality constraints were considered in [5] for nominal assets, and in [6] for real assets. Meanwhile, constraints given by quasi-concave inequalities are studied in [7]. Nevertheless, the most general approach remains to be the one in which restrictions are given by arbitrary closed and convex set containing the zero vector, as in [3] or [4], [2], [8], [9], [10] or [11].

Recently, the focus has been the study of restrictions that are affected by endogenous variables. [7] and [12] include consumption-price dependencies. Borrowing constraints depending on first-period consumption or wealth are considered, respectively, by [13] and [14]. A more general configuration is provided by [15], where the portfolio set depends on prices of commodities and assets.

In this paper, we analyze the existence of equilibria in incomplete financial markets when agents are subject to price-dependent trading constraints that affect the access to commodities and regulate financial trade. Two results of equilibrium existence are developed. First, we add investment constraints in [15], ensuring that a competitive equilibrium exists when individuals can fully hedge segmented assets payments by trading unsegmented contracts. Secondly, requiring weaker assumptions on preferences, we extend the model and the results of [13] allowing price-dependent trading constraints that affect the access to financial trade.

## Model

We focus on a two-period economy with a finite set of agents and uncertainty about the realization of a state of nature at the second period. Let S={0}∪S be the set of states of nature in the economy, where *s* = 0 denotes the unique state at the first period and *S* denotes the finite set of states that can be attained at the second period.

There is a finite number of commodities, which are perfectly divisible. Although previous results of the literature allow the transformation of commodities between periods, to simplify notation we focus on the case of perishable goods. However, our results can be easily extended to a framework with durable commodities. Commodity prices are denoted by $p={\left(p_{s}\right)}_{s\in S}\in R_{+}^{L\times S}$, where L is the set of commodities available for trade at each state of nature. Financial markets are composed by a finite set J of contracts and each j∈J is characterized by price-dependent contingent promises (*R*_*s*,*j*_(*p*))_*s*∈*S*_ which are continuous functions of prices and satisfy (*R*_*s*,*j*_(*p*))_*s*∈*S*_ ≠ 0 for every *p* ≫ 0. Prices of financial contracts are denoted by $q={\left(q_{j}\right)}_{j\in J}\in R_{+}^{J}$. Let $P≔R_{+}^{L\times S}\times R_{+}^{J}$ and $E≔R_{+}^{L\times S}\times R^{J}$ be, respectively, the space of prices and the set of admissible consumption bundles and financial portfolios.

There is a finite set of consumers I. Each agent *i* is characterized by a strictly increasing, strictly quasi-concave, and continuous utility function $V^{i}:R_{+}^{L\times S}\to R$, physical endowments $w^{i}={\left(w_{s}^{i}\right)}_{s\in S}\in R_{++}^{L\times S}$, and trading constraints determined by a correspondence $\Phi^{i}:P↠E$. Hence, given (p,q)∈P, each agent *i* maximizes her utility function by choosing consumption and financial positions in her choice set

$$C^{i}\left(p,q\right)≔\{\begin{array}{cc} \left(x^{i},z^{i}\right)\in \Phi^{i}\left(p,q\right): & p_{0}\cdot x_{0}^{i}+q\cdot z^{i}\leq p_{0}\cdot w_{0}^{i};\\ & p_{s}\cdot x_{s}^{i}\leq p_{s}\cdot w_{s}^{i}+\sum_{j\in J}R_{s,j}\left(p\right)z_{j}^{i},\;\;\;\forall s\in S. \end{array}\}.$$

This model is compatible with extreme cases of *financial market segmentation*, excluding agents of the financial trade of some assets, i.e.,

$$\{j\in J:\exists i\in I,\;\;\;\left(x^{i},z^{i}\right)\in \Phi^{i}\left(p,q\right)\;\Rightarrow z_{j}^{i}=0,\;\;\forall \left(p,q\right)\in P\}\neq \emptyset .$$

Also, we can allow *credit market exclusion*, as some agents may not have access to liquidity through financial contracts, i.e.,

$$\{i\in I:\;\;\left(x^{i},z^{i}\right)\in \Phi^{i}\left(p,q\right)\;\Rightarrow z^{i}\geq 0,\;\;\forall \left(p,q\right)\in P\}\neq \emptyset .$$

Definition 1. *A competitive equilibrium for the economy is a vector of prices*, *allocations*, *and portfolios* $\left(\left(\bar{p},\bar{q}\right),{\left({\bar{x}}^{i},{\bar{z}}^{i}\right)}_{i\in I}\right)\in P\times E^{I}$ *such that*:

(*i*) *Individual optimality*: $\left({\bar{x}}^{i},{\bar{z}}^{i}\right)\in \underset{\left(x^{i},z^{i}\right)\in C^{i}\left(\bar{p},\bar{q}\right)}{\text{argmax}}V^{i}\left(x^{i}\right),$ *for each* i∈I.
(*ii*) *Market feasibility*: $\sum_{i\in I}\left({\bar{x}}^{i},{\bar{z}}^{i}\right)=\sum_{i\in I}\left(w^{i},0\right).$

Under traditional assumptions on preferences and endowments, the difficulties that may appear to ensure equilibrium existence are associated with the effect of trading constraints on asset prices. Indeed, the restricted access to financial trade may increase the scarcity of instruments to transfer resources between periods. This situation may compromise the existence of upper bounds for asset prices and, therefore, our economy cannot be truncated to ensure equilibrium existence using standard fixed-point techniques.

These difficulties do not appear in the classical models without credit market segmentation. Indeed, when agents can always short-sale any financial contract, upper bounds on asset prices can be directly obtained by normalization of prices at the first-period. In our context, this normalization may compromise the well-behavior of choice set correspondences, inducing discontinuities on individual decisions. Indeed, since we do not require financial survival, i.e., that all agents have access to some amount of liquidity through any financial contract, after normalization of first-period prices, choice set correspondences may have an empty interior.

We conclude this section with some basic assumptions ensuring that the well-behavior of choice sets is not affected by trading constraints. The first of these hypotheses introduces some regularity conditions on trading constraints. To shorten notations, given j∈J, let ${\hat{e}}_{j}\in E$ be the plan composed by just one unit of investment on asset *j*.

Assumption A

(*i*) *For any* i∈I, $\Phi^{i}:P↠E$ *is lower hemicontinuous with convex values and closed graph*.
(*ii*) *For any* (p,q)∈P *and* i∈I, (0, 0) ∈ Φ^*i*^(*p*, *q*) *and* $\Phi^{i}\left(p,q\right)+\left(R_{+}^{L\times S}\times \left\{0\right\}\right)\subseteq \Phi^{i}\left(p,q\right)$.
(*iii*) *Given* (p,q)∈P *and* j∈J, *there is an agent* i∈I *such that* $\Phi^{i}\left(p,q\right)+{\hat{e}}_{j}\;\subseteq \;\Phi^{i}\left(p,q\right).$

Notice that, in contrast to [15], we may allow for restrictions on investment and, therefore, the property $\Phi^{i}\left(p,q\right)+R_{+}^{L\times S}\times R_{+}^{J}\subseteq \Phi^{i}\left(p,q\right)$ does not necessarily holds.

Restrictions on trading constraints are also imposed through assumptions over the correspondence of *attainable allocations* $\Omega :P↠E^{I}$, defined as the set-valued mapping that associates prices with market feasible allocations satisfying individuals’ budget and trading constraints, i.e.,

$$\Omega \left(p,q\right)≔\{{\left(\left(x^{i},z^{i}\right)\right)}_{i\in I}\in \prod_{i\in I}C^{i}\left(p,q\right):\sum_{i\in I}\left(x^{i},z^{i}\right)=\sum_{i\in I}\left(w^{i},0\right)\}.$$

Notice that, any element of Ω(*p*, *q*) satisfies budget constraints with equality.

Assumption B

*For any compact set* $P\subseteq R_{+}^{L\times S},\;\;\;\cup_{\left(p,q\right)\in P\times R_{+}^{J}:\;\left(p,q\right)\gg 0}\;\Omega \left(p,q\right)$ *is bounded*.

Boundedness conditions for the set of admissible consumptions and portfolios are already present in the literature. For instance, non-redundancy of asset payoffs induces this kind of requirements. Nevertheless, in a more general setting it should be interesting to consider some redundancy that arises when agents are restricted to participate in financial markets. Thus, Assumption B allows redundancy in assets markets as is already discussed in the Proposition and Example 4 by [15]. Our Assumption B is slightly stronger than the one required by [15] (Assumption A3) and it can be replaced only in our first result (Theorem 1) by a weaker one.

## Examples of trading constraints

In this section we show that our general approach to incomplete markets with trading constraints is compatible with the existence of security exchanges or assets backed by financial collateral.

### Example 1 (security exchanges)

Suppose that financial contracts are organized in *exchanges*, characterized by a partition of the sets of financial contracts $J=J_{1}\cup J_{2}\cup \cdots \cup J_{b}$. For each agent *i* let $G_{+}^{i},G_{-}^{i}:P↠\left\{J_{1},\ldots ,J_{b}\right\}$ be set-valued functions such that, for every (p,q)∈P,

$$\left(x^{i},z^{i}\right)\in \Phi^{i}\left(p,q\right)\;\;\Rightarrow \;\;\{\begin{array}{cc} z_{j}^{i}\geq 0, & \;\forall j\in G_{+}^{i}\left(p,q\right);\\ z_{j}^{i}\leq 0, & \;\forall j\in G_{-}^{i}\left(p,q\right);\\ z_{j}^{i}=0, & \;\forall j\notin G_{+}^{i}\left(p,q\right)\cup G_{-}^{i}\left(p,q\right). \end{array}$$

It follows that at prices (*p*, *q*), agent *i* can only short-sale assets in exchanges $G_{-}^{i}\left(p,q\right)$, whereas she can only invest in assets in exchanges $G_{+}^{i}\left(p,q\right)$. Also, the markets of debt and investment are not necessarily segmented, as $G_{+}^{i}\left(p,q\right)$ and $G_{-}^{i}\left(p,q\right)$ are not required to be disjoint.

Since the same agent can participate in several exchanges, we obtain a model of exchanges with heterogeneous participation, multi-membership, and price-dependent trading constraints. [16] address an equilibrium model with exchanges where individual preferences satisfy the kind of impatience condition imposed by [13]. Different to the example above, they allow cross-listing and transactions fees.

### Example 2 (financial collateral)

Since we allow for restrictions on investment, our model is compatible with the existence of assets backed by financial collateral. For instance, assume that there are financial contracts $j_{1},j_{2}\in J$ such that, for any (p,q)∈P we have that: $\left(x^{i},z^{i}\right)\in \Phi^{i}\left(p,q\right)\;\;\Rightarrow \;\;\text{max}\left\{z_{j_{2}}^{i},0\right\}\geq -\text{min}\left\{z_{j_{1}}^{i},0\right\}$ and $R_{s,j_{1}}\left(p\right)=\text{min}\left\{T_{s,j_{1}}\left(p\right),R_{s,j_{2}}\left(p\right)\right\},$ where $T_{s,j}:R_{+}^{L\times S}\to R_{+}$ is exogenously given.

Hence, each unit of asset *j*_1_ delivers an amount $T_{s,j_{1}}\left(p\right)$ at state of nature *s*, and it is backed by one unit of financial contract *j*_2_ in case of default. Notice that, as *j*_2_ serves as financial collateral, the investment in it may not be reduced without affecting trading feasibility.

These examples cannot be allowed in a model without investment restrictions. In the first one, the exclusion of financial markets allows to segment the set of financial contracts into separated exchanges. In the second one, the existence of financial collateral induce frictions in investment.

## Existence of Equilibrium

When traditional fixed-point techniques are used to prove the existence of a competitive equilibrium, one of the main steps is to ensure that endogenous variables can be bounded without adding frictions on the model. Since Assumption B induces endogenous bounds on market feasible allocations, we only need to find upper bounds for asset prices.

With this objective in mind, some authors impose *financial survival conditions*, assuming that every agent has access to resources by short-selling any financial contract (see [8], [10], [17] and [11]). Thus, commodity and asset prices can be normalized without compromising the lower hemicontinuity of individuals’ choice sets.

Notwithstanding, as we include financial market segmentation, we need to follow an alternative approach to establish bounds for asset prices. For this reason, we identify the set of assets that always give access to liquidity: we refer to a financial contract *j* as *unsegmented* when for every (p,q)∈P there is *δ* > 0 such that $-\delta {\hat{e}}_{j}\in \cap_{i\in I}\Phi^{i}\left(p,q\right)$. For notation convenience, let $J_{u}$ be the (possibly empty) maximal subset of J composed of contracts that are unsegmented.

Since any agent can short-sale unsegmented contracts, their prices can be normalized without affecting the continuity of individual demands. For this reason, we focus on hypotheses that allow us to find bounds for segmented asset prices: the super-replication of its deliveries by unsegmented contract promises or the compensation of losses on future consumption through the increment of current demand.

### Perfect-hedging of segmented contracts deliveries

In our first result of equilibrium existence we assume that promises of segmented contracts can be super-replicated by the deliveries of unsegmented contracts, an hypothesis that was imposed by [15] in a model without investment constraints.

Theorem 1. *Under Assumptions A and B*, *a competitive equilibrium exists when for every compact set* $P^{\prime }\subseteq P$ *there exists a portfolio of unsegmented asset* $\hat{z}\in R_{+}^{J_{u}}$ *that super-replicates the deliveries of segmented contracts*, *i.e*.,

$$\sum_{j\notin J_{u}}R_{s,j}\left(p\right)\leq \sum_{k\in J_{u}}R_{s,k}\left(p\right){\hat{z}}_{k},\;\;\forall s\in S,\;\forall \left(p,q\right)\in P^{\prime },$$

*and the following properties hold for any* $\left(p,q\right)\in P^{\prime }$, i∈I, *and* (*x*^*i*^, *z*^*i*^) ∈ Φ^*i*^(*p*, *q*),

(*i*) *Given* $j\in J_{u}$, $\left(x^{i},z^{i}\right)+{\hat{e}}_{j}\in \Phi^{i}\left(p,q\right)$.
(*ii*) *Given* $j\notin J_{u}$ *and* $\delta \in [0,\text{max}\left\{z_{j}^{i},0\right\}]$, $\left(x^{i},z^{i}\right)-\delta {\hat{e}}_{j}\in \Phi^{i}\left(p,q\right)$.

*Proof*. This result follows for almost identical arguments to those made in the proof of the main result of [15], CETM hereafter. Indeed, our hypotheses on utility functions, endowments, and trading constraints ensure the well-behavior of individuals’ best-reply correspondences and, therefore, there is an equilibrium in truncated economies in the sense of Lemma 2 of CETM. Furthermore, there are endogenous upper bounds for segmented asset prices, which can be obtained by following the arguments of Lemma 3 in CETM. This can be obtained by reducing investment in segmented assets without affecting the trading admissibility and the fact that all agents can increase the amount of investment on unsegmented assets, i.e., conditions (i) and (ii) in the statement of our Theorem. Finally, it follows from Lemma 4 in CETM and condition (i) that, for sufficiently large upper bounds on asset prices, the set of equilibria in truncated economies coincides with the set of competitive equilibria.

Comparing Theorem 1 with the main result of [15], it follows that the inclusion of trading constraints affecting the trade of long-positions on financial contracts does not compromises equilibrium existence provided condition (i) above holds.

### Impatience on preferences

The second approach to equilibrium existence extends [13] in order to include price-dependent trading constraints and investment restrictions. Thus, we ensure the compatibility between equilibrium without requiring the fully hedging of segmented asset promises and, therefore, allowing the exclusion of financial markets as the set of unsegmented contracts can be empty.

We also guarantee that the main result of [13] holds under weaker assumptions. In fact, we only impose their impatience condition on a subset of agents. More importantly, in [13] it is assumed that sets of trading admissible short-sales are compact, a hypothesis that is stronger than the requirements of the next result.

Theorem 2. *Under Assumptions A and B*, *there exists a competitive equilibrium if there is a non-empty subset of agents* $I^{*}\subseteq I$ *such that*:

(*i*) *Given* $i\in I^{*}$ *and* $\left(\rho ,x^{i}\right)\in \left(0,1\right)\times R_{++}^{L\times S}$, *there exists* $\tau^{i}\left(\rho ,x^{i}\right)\in R_{+}^{L}$ *such that*, $\;V^{i}(x_{0}^{i}+\tau^{i}\left(\rho ,x^{i}\right),{\left(\rho \;x_{s}^{i}\right)}_{s\in S})>V^{i}\left(x^{i}\right).$
(*ii*) *Given* $j\notin J_{u}$, *there is* $i\in I^{*}$ *and* $z^{i}\in {\mathbb{R}}_{-}^{J}$ *such that* $z_{j}^{i}<0$ *and* $\left(0,z^{i}\right)\in \Phi^{i}\left(p,q\right),\;\forall \left(p,q\right)\in P$.

The proof is given in Appendix A.

The requirement (i) in Theorem 2 holds independently of the representation of preferences, and was introduced by [13] to analyze equilibrium existence in a model with borrowing constraints depending on first-period consumption ([18] extended the results of [13] including price-dependent trading constraints in an environment with non-ordered preferences. As in [13], it is assumed that correspondences of trading admissible allocations have compact values). Intuitively, it requires the existence of agents that, in terms of preferences, can compensate any loss in well-being associated with a reduction in future demand with an increment of present consumption. The requirement (ii) in Theorem 2 guarantees that, for any segmented contract, and independently of prices, there is at least one agent that can short-sale it without the need to invest on other assets or to consume commodities. In particular, the conditions required in Theorem 2 are satisfied when there is an agent *h* such that *V*^*h*^ is unbounded on first-period consumption and, independent of prices (p,q)∈P, the zero vector belongs to the interior of Φ^*h*^(*p*, *q*).

In this context, the main idea behind the existence of upper bounds for asset prices is as follows: consider an agent $i\in I^{*}$ such that, at prices (p,q)∈P, her optimal consumption allocation is market feasible. Suppose that, as an alternative to her optimal strategy, she decides to make a promise on an asset $j\notin J_{u}$ using the borrowed resources to increase first-period consumption. Also, assume that this promise can be paid with her future endowments. As a consequence, if the new strategy generates a high enough liquidity, then she will ensure a utility level greater than the one associated to aggregated endowments. A contradiction with the market feasibility of her optimal allocation. Thus, *q*_*j*_ needs to be bounded (see Lemma 1 in Appendix A for detailed arguments).

Corollary. *Under Assumptions A and B*, *assume that there is a set of agents* $I^{⋄}\subseteq I$ *satisfying condition (ii) of Theorem 2 such that*,

$$\forall i\in I^{⋄},\;\exists l\in L:\;\;V^{i}\left(x^{i}\right)=v_{l}^{i}\left(x_{0,l}^{i}\right)+v^{i}\left({\left(x_{0,r}^{i}\right)}_{r\neq l},{\left(x_{s}^{i}\right)}_{s\in S}\right),$$

*where* $v_{l}^{i}:R_{+}\to R$ *is a concave function*. *Then*, *there exists a competitive equilibrium for the economy with endogenous trading constraints*.

The proof is given in Appendix B.

Essentially, when there are agents whose utility functions satisfy the separability condition above, we can construct an auxiliary economy where these individuals have preferences satisfying the requirement (i) in Theorem 2. Thus, we may obtain equilibrium existence by applying our previous result and showing that any equilibrium of the auxiliary economy is an equilibrium of the original one. This corollary is inspired by a result of [19] (Corollary 2) for equilibrium existence in infinite horizon incomplete markets economies.

## Concluding Remarks

We provide a general framework for two-period economies with uncertainty where restricted access to markets is considered. We extend the literature by allowing a general form of constraints in consumption and portfolios that we called trading constraints in the spirit of [15]. In the one hand, we include price dependence in trading contraints generalizing configurations where restrictions are exogenous to the model. In the other hand, we include frictions to investment opportunities that were not present before in the context of endogenous trading constraints.

Our results combine two different alternatives to ensure equilibrium existence. The first one, requiring perfect hedging of the financial structure that is segmented. The second, by assuming a kind of impatience condition in agents’ preferences.

Some advantages of our approach are the generality of the configuration that includes several types of financial contracts, e.g. wealth-dependent access, borrowing constraints, commodity options, or financial collateral, and to dispense of differentiability assumptions over trading restrictions.

This general configuration is interesting, in particular, because if they are complemented with weaker conditions over the access to the market, then segmentation and/or exclusion of markets are present in the model.

As a matter of future research, there are different directions to take. A first one that study the relation between inter-temporal transfers and endogenous bounds for prices imposed by super-replication or impatience in preferences. A second one exploring time variations of the trading constraints, for instance, in order to consider credit contractions in the spirit of [20]. A third way may include structures justifying the formation of exchanges in terms of networks or group formation.

## Appendix A: Proof of the Theorem 2

For each M∈N, let $P\left(M\right)≔P\times {\left[0,M\right]}^{J\setminus J_{u}}\subseteq P$ where

$$P=\{\left({\left(p_{s}\right)}_{s\in S},{\left(q_{j}\right)}_{j\in J_{u}}\right)\in R_{+}^{L\times S}\times R_{+}^{J_{u}}:∥\left(p_{0},{\left(q_{j}\right)}_{j\in J_{u}}\right){∥}_{\Sigma }=1\;\wedge \;∥p_{s}{∥}_{\Sigma }=1,\;\forall s\in S\}.$$

Given *N* > 0, define $K\left(N\right)≔[0,2\hat{W}+N{]}^{L\times S}\times [-\hat{\Omega },\;\#I\;\hat{\Omega }{]}^{J},$ where

$$\hat{W}≔(\#J\;\#I\;\;\hat{\Omega }+\sum_{(s,l)\in S\times L}\;\;\sum_{i\in I}w_{s,l}^{i})(1+\underset{s\in S}{\text{max}}\;\underset{(p,q)\in P}{\text{max}}\;\sum_{j\in J}\;R_{s,j}\left(p\right)),$$

$$\hat{\Omega }≔2\underset{\left(p,q\right)\in P\times R_{+}^{J\setminus J_{u}}:\;\left(p,q\right)\gg 0}{\text{sup}}\;\;\;\;\underset{{\left(x^{i},z^{i}\right)}_{i\in I}\in \Omega \left(p,q\right)}{\text{sup}}\;\;\;\sum_{i\in I}\;{∥z^{i}∥}_{\Sigma }.$$

Notice that, Assumption C guarantees that $\hat{\Omega }$ is finite.

Let $\Psi_{M,N}:P\left(M\right)\times {\left(K\left(N\right)\right)}^{I}↠P\left(M\right)\times {\left(K\left(N\right)\right)}^{I}$ be the correspondence given by

$$\Psi_{M,N}\left(p,q,{\left(x^{i},z^{i}\right)}_{i\in I}\right)=\phi_{M}\left({\left(x^{i},z^{i}\right)}_{i\in I}\right)\times \prod_{i\in I}\phi_{N}^{i}\left(p,q\right),$$

where

$$\begin{array}{ccc} \phi_{M}\left({\left(x^{i},z^{i}\right)}_{i\in I}\right) & ≔ & \underset{\left(p,q)\in P(M\right)}{\text{argmax}}\;\;\sum_{s\in S}p_{s}\cdot \sum_{i\in I}\left(x_{s}^{i}-w_{s}^{i}\right)+q\cdot \sum_{i\in I}z^{i},\\ \phi_{N}^{i}\left(p,q\right) & ≔ & \underset{\left(x^{i},z^{i}\right)\in C^{i}\left(p,q\right)\cap K\left(N\right)}{\text{argmax}}\;V^{i}\left(x^{i}\right),\;\forall i\in I. \end{array}$$

It follows from identical arguments to those given in [15] (Lemmata 1 and 2) that for each (*M*, *N*) ≫ 0 the correspondence Ψ_*M*,*N*_ has a non-empty set of fixed points. Therefore, our objective is to ensure that, for *M* and *N* large enough the fixed points of Ψ_*M*,*N*_ are competitive equilibria for our economy. Hence, we need to determine upper bounds for prices of segmented assets.

Lemma 1. *Under the Assumptions of Theorem 2*, *let* $\left(\bar{p},\bar{q},{\left({\bar{x}}^{i},{\bar{z}}^{i}\right)}_{i\in I}\right)$ *be a fixed point of* Ψ_*M*,*N*_ *satisfying* ${\bar{x}}_{s,l}^{i}<2\hat{W},\;\;\;\forall \left(s,l\right)\in S\times L.$ *Then*, *there is* $\hat{Q}>0$ *such that*, *for N large enough*, ${\bar{q}}_{j}\leq \hat{Q},\;\;\forall j\notin J_{u}.$

Proof. For any $i\in I^{*}$, let *ρ*^*i*^ ∈ (0, 1) such that $2\hat{W}\rho^{i}=0.25\underset{(s,l)\in S\times L}{\text{min}}w_{s,l}^{i}$. Hence, property (i) of Theorem 2 imply that

$$V^{i}\left({\bar{x}}^{i}\right)\leq V^{i}\left(2\hat{W}\left(1,\ldots ,1\right)\right)<V^{i}(2\hat{W}\left(1,\ldots ,1\right)+\tau^{i}\left(\rho^{i},2\hat{W}\left(1,\ldots ,1\right)\right),(\frac{w_{s}^{i}}{2}{)}_{s\in S}).$$

Fix $j\notin J_{u}$ and $i=i\left(j\right)\in I^{*}$ satisfying part (ii) of Theorem 2. Then, there is *z*^*i*^ ≤ 0 such that $z_{j}^{i}<0$ and $\left(0,z^{i}\right)\in \Phi^{i}\left(p,q\right),\;\;\forall \left(p,q\right)\in P$. Since Φ^*i*^ has convex values and $\left(0,0\right)\in \Phi^{i}\left(\bar{p},\bar{q}\right)$, it follows that $\left(0,\varepsilon z^{i}\right)\in \Phi^{i}\left(\bar{p},\bar{q}\right),\;\forall \varepsilon \in \left[0,1\right]$. Also, the continuity of payoffs ensures that there is *ε*^*i*^ ∈ (0, 1) such that,

$$\varepsilon^{i}\;\underset{(p,q)\in P}{\text{max}}\;\;\underset{s\in S}{\text{max}}\;\;\sum_{k\in J}\;R_{s,k}\left(p\right)\;z_{k}^{i}<0.5\underset{(s,l)\in S\times L}{\text{min}}w_{s,l}^{i}.$$

In addition, it follows from Assumption A(ii) that, for each $N>\hat{N}≔\underset{i\in I^{*}}{\text{max}}\;{∥\tau^{i}\left(\rho^{i},2\hat{W}\left(1,\ldots ,1\right)\right)∥}_{\Sigma }$

$$((2\hat{W}\left(1,\ldots ,1\right)+\tau^{i}\left(\rho^{i},2\hat{W}\left(1,\ldots ,1\right)\right),(\frac{w_{s}^{i}}{2}{)}_{s\in S}),\;\varepsilon^{i}z^{i}\;)\;\;\in \;\;\Phi^{i}\left(\bar{p},\bar{q}\right)\cap \hat{K}\left(N\right).$$

Consequently, as $\left({\bar{x}}^{i},{\bar{z}}^{i}\right)$ is an optimal choice for agent *i* in $C^{i}\left(\bar{p},\bar{q}\right)\cap \hat{K}\left(N\right)$ and *z*^*i*^ ≤ 0, it follows that

$$2\hat{W}∥{\bar{p}}_{0}{∥}_{\Sigma }+{\bar{p}}_{0}\cdot \left(\tau^{i}\left(\rho^{i},2\hat{W}\left(1,\ldots ,1\right)\right)-w_{0}^{i}\right)>-\varepsilon^{i}\;\bar{q}\cdot z^{i}\geq \varepsilon^{i}{\bar{q}}_{j}\left|z_{j}^{i}\right|,$$

which implies that ${\bar{q}}_{j}\leq \left(2\hat{W}+\hat{N}\right)/(\varepsilon^{i}|z_{j}^{i}\left|\right).$ Since *i* = *i*(*j*) was fixed, we can consider

$$\hat{Q}≔\underset{j\notin J_{u}}{\text{max}}\;\;\frac{2\hat{W}+\hat{N}}{\varepsilon^{i(j)}\left|z_{j}^{i(j)}\right|}.$$

Lemma 2. *Under the Assumptions of Theorem 2*, *fix* $\left(M,N\right)\gg \left(\hat{Q},\hat{N}\right).$ *Then*, *each fixed point of* Ψ_*M*,*N*_ *is a competitive equilibrium of our economy*.

This result follows from identical arguments to those made in the proof of Lemma 4 in [15].

## Appendix B: Proof of the Corollary

For each agent $i\in I^{⋄}$, let ${\tilde{V}}^{i}:R_{+}^{L\times S}\to R$ be the function defined by

$${\tilde{V}}^{i}\left(x^{i}\right)=v_{l(i)}^{i}(\text{min}\{x_{0,l(i)}^{i},2W_{0,l(i)}\})+\rho^{i}\text{max}\{x_{0,l(i)}^{i}-2W_{0,l(i)},\;0\}+v^{i}\left({\left(x_{0,r}^{i}\right)}_{r\neq l(i)},\;{\left(x_{s}^{i}\right)}_{s\in S}\right),$$

where l(i)∈L is the commodity that satisfies the condition of the Corollary, $W_{0,l(i)}=\sum_{h\in I}w_{0,l(i)}^{h}$, and $\rho^{i}\in \partial v_{l(i)}^{i}\left(2W_{0,l(i)}\right)$. As customary, $\partial v_{l(i)}^{i}\left(x\right)≔\left\{\rho \in R:v_{l(i)}^{i}\left(y\right)-v_{l(i)}^{i}\left(x\right)\leq \rho \left(y-x\right),\;\forall y\geq 0\right\}$ denotes the super-differential of $v_{l(i)}^{i}$ at point *x*. Notice that, as $W_{0}≔{\left(W_{0,l}\right)}_{l\in L}\gg 0$, the monotonicity and concavity of $v_{l(i)}^{i}$ ensure that $\partial v_{l(i)}^{i}\left(2W_{0,l(i)}\right)$ is a non-empty subset of $R_{+}$.

Consider the economy obtained by replacing ${\left\{V^{i}\right\}}_{i\in I^{⋄}}$ with ${\left\{{\tilde{V}}^{i}\right\}}_{i\in I^{⋄}}$. Then, part (i) of Theorem 2 holds. Hence, Theorem 2 guarantees that there exists a competitive equilibrium $\left(\left(\bar{p},\bar{q}\right),{\left({\bar{x}}^{i},{\bar{z}}^{i}\right)}_{i\in I}\right)\in P\times E^{I}$ for this auxiliary economy. To conclude the proof it is sufficient to ensure that $\left({\bar{x}}^{i},{\bar{z}}^{i}\right)$ satisfies $V^{i}\left({\bar{x}}^{i}\right)\geq V^{i}\left(x^{i}\right),\;\forall i\in I^{⋄},\;\forall \left(x^{i},z^{i}\right)\in C^{i}\left(\bar{p},\bar{q}\right)$.

Suppose, by contradiction, that there exists $i\in I^{⋄}$ and $\left(x^{i},z^{i}\right)\in C^{i}\left(\bar{p},\bar{q}\right)$ such that $V^{i}\left(x^{i}\right)>V^{i}\left({\bar{x}}^{i}\right)$. The market feasibility of consumption allocations ensures that, for every l∈L, ${\bar{x}}_{0,l}^{i}<2W_{0,l}$. Therefore, ${\tilde{V}}^{i}\left({\bar{x}}^{i}\right)=V^{i}\left({\bar{x}}^{i}\right)$ and there exists λ ∈ (0, 1) such that $\lambda {\bar{x}}_{0,l}^{i}+\left(1-\lambda \right)x_{0,l}^{i}<2W_{0,l},\forall l\in L$.

Since $\lambda \left({\bar{x}}^{i},{\bar{z}}^{i}\right)+\left(1-\lambda \right)\left(x^{i},z^{i}\right)\in C^{i}\left(\bar{p},\bar{q}\right)$, we conclude that,

$${\tilde{V}}^{i}\left({\bar{x}}^{i}\right)=V^{i}\left({\bar{x}}^{i}\right)=\text{min}\left\{V^{i}\left({\bar{x}}^{i}\right),V^{i}\left(x^{i}\right)\right\}<V^{i}\left(\lambda {\bar{x}}^{i}+\left(1-\lambda \right)x^{i}\right)={\tilde{V}}^{i}\left(\lambda {\bar{x}}^{i}+\left(1-\lambda \right)x^{i}\right).$$

This contradicts the optimality of $\left({\bar{x}}^{i},{\bar{z}}^{i}\right)$ for agent *i* in the auxiliary economy.

## References

[1] Radner R, Existence of equilibrium of plans, prices, and price expectations. *Econometrica* 1972 40:289–303. 10.2307/1909407

[2] Siconolfi P. Equilibrium with asymmetric constraints on portfolio holdings and incomplete financial markets *Non-Linear Dynamics in Economics and Social Sciences*, Galeotti M., Geronazzo L., Gori F. editors, Societa’ Pitagora 1989 271–292.

[3] Cass D Competitive equilibrium with incomplete financial markets. CARESS working paper 1984

[4] Cass D Competitive equilibrium with incomplete financial markets. *J Math Econ*. 2006 42:384–405. 10.1016/j.jmateco.2006.04.008

[5] Balasko Y, Cass D, Siconolfi P. The structure of financial equilibrium with endogenous yields. *J Math Econ*. 1990 19:195–216. 10.1016/0304-4068(90)90042-8

[6] Polemarchakis H, Siconolfi P. Generic existence of competitive equilibria with restricted participation. *J Math Econ*. 1997 28:289–311. 10.1016/S0304-4068(96)00802-6

[7] Cass D, Siconolfi P, Villanacci A. Generic regularity of competitive equilibria with restricted participation. *J Math Econ*. 2001 45:787–806.

[8] Angeloni L, Cornet B. Existence of financial equilibria in a multi-period stochastic economy. *Advances in Mathematical Economics*. 2006 8:933–955.

[9] Cornet B, Gopalan R. Arbitrage and equilibrium with portfolio constraints. *Econ Theor*. 2010 45:227–252. 10.1007/s00199-009-0506-5

[10] Aouani Z, Cornet B: Existence of financial equilibria with restricted participation. *J Math Econ*. 2009 45:772–786. 10.1016/j.jmateco.2009.10.001

[11] Aouani Z, Cornet B. Reduced equivalent form of a financial structure. *J Math Econ*. 2011 47:318–327. 10.1016/j.jmateco.2010.12.015

[12] Carosi L, Gori M, Villanacci A. Endogenous restricted participation in general financial equilibrium. *J Math Econ*. 2009 45:787–806 10.1016/j.jmateco.2009.06.008

[13] Seghir A, Torres-Martínez JP On equilibrium existence with endogenous restricted financial participation. *J Math Econ*. 2011 47:37–42. 10.1016/j.jmateco.2010.10.006

[14] Hoelle M, Pireddu M, Villanacci A. Incomplete Financial Markets with Real Assets and Wealth-Dependent Credit Limits. *J Econ* 2016 117:1–36. 10.1007/s00712-015-0438-4

[15] Cea-Echenique S, Torres-Martínez JP Credit segmentation in general equilibrium. *J. Math Econ*. 2016 62:19–27. 10.1016/j.jmateco.2015.10.011

[16] Faias M, Luque J. Cross-listed Securities and Multiple Exchange Memberships: Demand Differentiability and Equilibrium Existence. *BE J Theor Econ*. 2017 18:1935–1704

[17] Hahn G, Won DC. Equilibrium in financial markets with market frictions. *The Korean Econ Rev* 2007 23:267–302.

[18] Pérez-Fernández V. Incomplete financial participation: exclusive markets, investment clubs and credit risk. Working paper 2013.

[19] Moreno-García E, Torres-Martínez JP. Equilibrium existence in infinite horizon economies. *Port Econ J*. 2012 11:127–145. 10.1007/s10258-012-0079-2

[20] Iraola MA, Torres-Martínez JP. Equilibrium in collateralized asset markets: credit contractions and negative equity loans. *J Math Econ*. 2014 55:113–122. 10.1016/j.jmateco.2014.10.006

